# Tubulointerstitial Nephritis With Uveitis (TINU) Syndrome: A Case Series and Review of Literature

**DOI:** 10.1111/nep.70271

**Published:** 2026-08-27

**Authors:** Weaam Ali, Nidhi Agrawal, Smeeta Sinha, Dimitrios Poulikakos, Jamil Choudhury, Constantina Chrysochou

**Affiliations:** ^1^ Nephrology Department, Salford Royal Hospital Northern Care Alliance Salford Greater Manchester UK; ^2^ Donal O'Donoghue Renal Research Centre Northern Care Alliance Salford Greater Manchester UK; ^3^ Division of Cardiovascular Sciences, Faculty of Biology, Medicine & Health The University of Manchester Manchester UK; ^4^ Histopathology Department, Salford Royal Hospital Northern Care Alliance Salford Greater Manchester UK

**Keywords:** chronic kidney disease, relapsing disease, TINU syndrome, tubulointerstitial nephritis, uveitis

## Abstract

Tubulointerstitial nephritis with uveitis (TINU) syndrome is a rare disorder characterised by the simultaneous or sequential occurrence of acute tubulointerstitial nephritis and uveitis, in the absence of systemic disease. Its true prevalence is likely underestimated, as renal and ocular manifestations may not appear concurrently. We conducted a retrospective case series of patients diagnosed with TINU at a single tertiary centre between 2016 and 2025. Clinical, biochemical, histopathological and ophthalmological data were reviewed. Long‐term renal and ocular outcomes were assessed. Ten patients (female:male 6:4) were identified, with a median age at diagnosis of 41 years (range 15–67). Renal disease preceded uveitis in 60% of cases, with a mean interval of 5.6 months. At presentation, 70% had serum creatinine > 200 μmol/L and one patient required transient dialysis. All renal biopsies demonstrated tubulointerstitial nephritis with preserved glomeruli. All patients were treated with systemic corticosteroids, with topical therapy for uveitis. Renal relapse occurred in 60% of patients, often during steroid tapering, and required prolonged corticosteroid therapy or mycophenolate mofetil. At final follow‐up (median 44 months), 50% had chronic kidney disease (eGFR < 60 mL/min). Relapsing disease was more frequent in females and patients under 18 years. At last follow‐up, mean serum creatinine was 89 μmol/L, with 50% of patients having CKD G3a. Ophthalmologic data (*n* = 6) showed bilateral anterior uveitis in all cases, with intermediate involvement in three. Two developed steroid‐induced intraocular hypertension. Ocular relapse occurred in six patients and frequently paralleled renal disease activity. We herein report one of the longest median follow‐up durations (44 months) among biopsy‐proven cohorts of interstitial nephritis. Our cohort spans a broader age range (15–67 years) than traditionally described with an adult predominance. Despite a 60% relapse rate, response to steroids and immunosuppression was good. Proteinuria at presentation was noted in individuals who developed subsequent CKD supporting the need for follow up and proteinuria management where relevant.

## Summary at a Glance

This case series describes 10 patients with biopsy‐proven tubulointerstitial nephritis with uveitis (TINU) and long‐term follow‐up. Renal and ocular relapse occurred in 60% of cases and was often synchronous, with progression to chronic kidney disease in half of the patients. Early recognition and prolonged follow‐up are essential.

## 1 Introduction

Tubulointerstitial nephritis with uveitis (TINU) syndrome is an uncommon entity characterised by simultaneous or successive involvement of the eyes and kidneys in the form of uveitis and acute tubulointerstitial nephritis, respectively, in the absence of any systemic disease. TINU accounts for approximately 5% of cases of interstitial nephritis and around 2% of cases seen in uveitis clinics [1, 2]. However, these figures may not reflect the true prevalence of TINU because it is likely underdiagnosed, as renal and ocular involvement do not always occur concurrently. Renal disease may also be overlooked if renal function is only mildly impaired and not evaluated. The condition was first described by Dobrin et al. in 1975 [3], who reported two patients with anterior uveitis, renal failure, bone marrow granulomas and hypogammaglobulinemia who did not meet diagnostic criteria for any other systemic illness.

## 2 Methods

This was an observational case series conducted at a single tertiary care centre. Data were collected using electronic patient records of patients diagnosed with TINU syndrome. Both newly diagnosed patients and those already under follow‐up during the study period were included.

Renal outcomes were assessed using serum creatinine, estimated glomerular filtration rate (eGFR) and urine protein–creatinine ratio (uPCR). Ophthalmologic characteristics, treatment, and relapse data were obtained from specialist clinic documentation. Outcomes of interest included renal and ocular response to therapy, relapse rates, requirement for second‐line immunosuppression, and progression to chronic kidney disease.

Descriptive statistics were used to summarise demographic, clinical, laboratory, histopathological and ophthalmologic variables.

## 3 Cases Review

We present a case series of 10 patients with TINU diagnosed between January 2016 and November 2025 via renal biopsy who remain under follow‐up. The median follow‐up duration was 44 months. There was a female preponderance (female: male = 6:4). Median age at presentation was 41 years (range 15–67). Six (60%) patients presented initially with renal disease and later developed uveitis, while four (40%) presented with concurrent renal and ocular involvement. Among those who developed uveitis after kidney disease, the mean time to ocular diagnosis was 5.6 months. Seven (70%) patients had a serum creatinine level > 200 μmol/L at presentation; one required two sessions of dialysis. Urine dipstick data were available for seven patients, of whom five showed hematuria and proteinuria. The median uPCR at presentation was 100 mg/mmol, and only one patient had nephrotic‐range proteinuria. No specific precipitating factor was identified, although one patient reported NSAID use prior to onset. There was no evidence of COVID‐19 infection in the patients who were diagnosed after the onset of the COVID‐19 pandemic.

**FIGURE 1:**
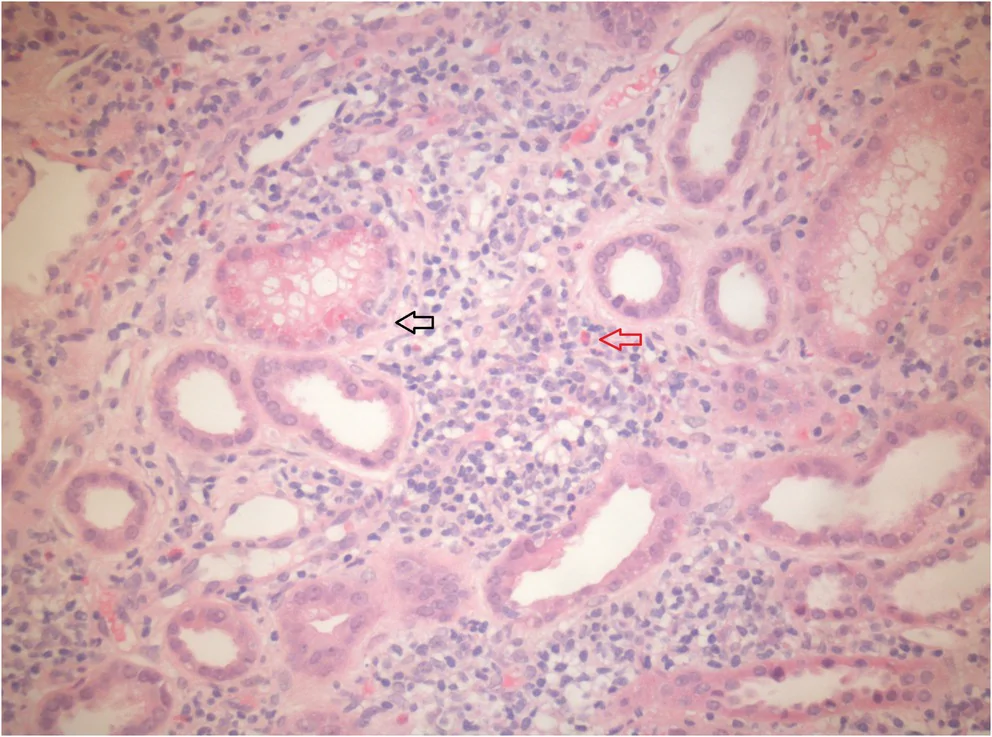
Renal biopsy showing tubulointerstitial nephritis, with lymphocytes, plasma cells, eosinophils (red arrow) and occasional mild tubulitis (black arrow). Eosinophils may be present in TINU as part of the acute inflammatory infiltrate and should be interpreted in the appropriate clinicopathological context.

Hypergammaglobulinemia was present in six (60%) patients, with all immunoglobulin subclasses (IgG, IgA and IgM) raised in those < 20 years of age. ACE levels were normal in all seven patients with available results. Autoimmune serology (ANA, ANCA and complements) was negative except in one 15‐year‐old female with positive anti‐chromatin antibodies. All patients underwent renal biopsy, which demonstrated features of tubulointerstitial nephritis with normal glomeruli (Figure 1).

All patients received oral prednisolone as first‐line therapy. The initial dose was typically 60 mg daily, followed by gradual tapering according to clinical response, generally by 5 mg every 2 weeks. The overall duration of corticosteroid therapy varied between patients and is summarised in Table 1. Mycophenolate mofetil (MMF) was used as a steroid‐sparing agent in patients with relapsing disease or where prolonged immunosuppression was required. MMF was typically initiated at a dose of 1 g twice daily. In most cases, MMF was introduced during corticosteroid tapering and continued after steroid discontinuation, although a small number of patients received MMF monotherapy following relapse. Treatment duration varied according to clinical response and is detailed in Table 1. To further characterise treatment response, a comparison was performed between patients managed with corticosteroids alone and those requiring MMF. Patients receiving MMF were generally older, experienced more frequent relapses and required longer treatment durations, although renal outcomes at latest follow‐up were broadly similar between groups (Table 2).

**TABLE 1:** Summary of clinical characteristics and treatment characteristics of patients in our TINU cohort.

Patient	Age (years) at diagnosis	Gender	Creatinine at presentation (μmol/L)	Latest serum creatinine (μmol/L)	eGFR at presentation (mL/min/1.73 m^2^)	Latest eGFR (ml/min/1.73 m^2^)	Upcr at presentation (mg/mmol)	Urine dipstick at presentation	Immunoglobulins (IgA, IgM, IgG)
1	15	F	175	71	38	> 90	35	NA	All raised
2	15	M	218	79	39	> 90	89	NA	All raised
3	16	F	702	70	7	> 90	87	NA	All raised
4	21	M	370	96	17	83	65	Blood++, protein trace	All raised
5	31	F	232	103	21	53	141	Negative	Normal
6	51	F	690 (required 2× of dialysis)	88	5	58	378	Hematoprotienuria	Raised IgG
7	54	F	360	75	12	69	124	Hematoprotienuria	Raised IgA
8	59	M	216	132	26	47	16	Protein trace	Normal
9	63	M	172	167	35	36	211	Protein++++, blood++	Normal
10	67	F	151	104	31	46	112	Protein+++, blood+	Normal

Patient	Immunology screen	Degree of IFTA on biopsy	Organ involved first	Renal relapse	MMF use	Treatment duration	Ocular disease type	Time to ocular involvement (months)	Ocular relapse
1	Anti chromatin pos, beta 2 micrglob pos, high c3	NA	Concurrent	Yes	Yes	15 months	Anterior uveitis	NA	Yes
2	Negative	NA	Kidney	Yes	No	8 months	Anterior uveitis	6	Yes
3	Negative	NA	Kidney	Yes	Yes	43.2 months	Anterior + intermediate uveitis	6	Yes
4	Negative	Mild	Kidney	No	No	3 months	Anterior uveitis	Uknown	No
5	Negative	20% IFTA	Concurrent	No	No	2 months	Iritis	NA	Yes
6	Negative	Mild	Kidney	Yes	Yes	40.8 months	Anterior and intermediate uveitis	8	No
7	Negative	NA	Kidney	No	No	5 months	Anterior uveitis	5	No
8	Negative	Moderate	Concurrent	No	No	4 months	Uveitis	NA	Yes
9	Negative	25% IFTA	Kidney	Yes	Yes	11 months	Anterior uveitis	3	No
10	Negative	50% IFTA	Concurrent	Yes	Yes	11 months	Anterior uveitis	NA	Yes

*Note:* This table summarises demographic data, renal parameters at presentation, immunologic findings, patterns of organ involvement, ocular disease characteristics, treatment received and clinical outcomes for all patients diagnosed with tubulointerstitial nephritis and uveitis (TINU) in our cohort.

Total treatment duration includes initial corticosteroid therapy and any subsequent relapse treatment, including MMF where applicable. Treatment duration for patients still receiving therapy is reported up to the latest follow‐up.

The 2021 CKD‐EPI creatinine equation was used to estimate GFR in patients aged < 18 years to allow comparison across the cohort.

Abbreviations: eGFR = estimated glomerular filtration rate, F = female, IFTA = interstitial fibrosis and tubular atrophy, Ig = immunoglobulin, M = male, MMF = mycophenolate mofetil, NA = not applicable, UPCR = urine protein‐to‐creatinine ratio.

**TABLE 2:** Comparison of patients treated with corticosteroids alone and those requiring mycophenolate mofetil (MMF).

Characteristic	Corticosteroids alone (*n* = 5)	MMF group (*n* = 5)
Median age (years) (range)	31 (15–59)	51 (15–67)
Female sex, *n* (%)	2 (40)	4 (80)
Mean serum creatinine at presentation (μmol/L)	285 ± 119	378 ± 290
Median treatment duration (months) (range)	4 (2–6)	15 (11–43)
CKD at final follow‐up (eGFR < 60 mL/min/1.73 m^2^), *n* (%)	2 (40)	3 (60)
Mean latest serum creatinine (μmol/L)	97 ± 23	100 ± 40
Mean latest eGFR (mL/min/1.73 m^2^)	68	64

*Note:* Data are presented as median (range), mean ± standard deviation or number (%), as appropriate. MMF was prescribed for patients with relapsing or steroid‐dependent disease; therefore, the two groups are not directly comparable and the analysis is descriptive only.

Six (60%) patients experienced renal relapse during tapering or soon after steroid cessation and required either prolonged corticosteroids or MMF as a second‐line agent. Duration of relapse treatment ranged from 3 months to 3 years based on clinical response. Relapsing disease occurred predominantly in females (75%) and in younger patients (< 18 years). At the latest follow‐up, the mean serum creatinine was 89 μmol/L. All patients with available data had uPCR < 30 mg/mmol except one patient with uPCR of 77 mg/mmol. Five patients developed CKD (eGFR < 60 mL/min), including one aged under 50 years (34‐year‐old female). The patient who required dialysis initially had a current eGFR of 58 mL/min.

### 3.1 Ophthalmologic Findings

Detailed ophthalmology data were available for six patients. All had bilateral involvement, predominantly anterior uveitis; three had additional intermediate uveitis. Three presented symptomatically with red, painful eyes and reduced vision, while the remainder were diagnosed incidentally during screening. All were treated initially with topical corticosteroids. Six experienced ocular relapse following cessation of topical therapy, necessitating prolonged treatment (2–10 months). Raised intraocular pressure occurred in two patients during corticosteroid therapy and was managed conservatively. Overall, ocular inflammation responded well to topical and systemic corticosteroids, with flares often coinciding with renal relapse.

## 4 Discussion

Mandeville and Foster [4] reviewed 133 cases of TINU reported worldwide and established diagnostic criteria based on biopsy‐proven tubulointerstitial nephritis and characteristic uveitis occurring within a defined temporal relationship. Regusci et al. [5] reviewed 592 reported cases and found that TINU predominantly affects adolescents, with a female predominance and bilateral anterior uveitis as the most common ocular manifestation. Initial renal involvement followed by uveitis was the most common presentation followed by concurrent involvement. Ocular relapses were more common than renal relapses and were predominantly seen in children. A very few patients (*n* = 18) required haemodialysis. 15% of patients had autoantibodies (ANA and ANCA) and it was significantly higher in females. Most of the patients were treated with steroids (systemic and/or topical) and around one‐fifth of patients required second line therapy. Progression to CKD was more frequent in adults. Posterior or panuveitis and older age were found to have increased risk of progressing to CKD.

The characteristics of our cohort were quite similar to those reported in the literature. In our cohort, the lowest eGFR values at follow‐up were observed in the oldest patients. Six patients had evidence of IFTA on biopsy, including three with moderate IFTA, all aged over 50 years, suggesting that chronic tubulointerstitial damage may have contributed to poorer renal outcomes. However, age‐related decline in kidney function, delayed diagnosis and vascular comorbidities may also have contributed, and these factors could not be distinguished in this retrospective study.

Pathogenesis of TINU is believed to be autoimmune with both cell‐mediated and humoral immune responses implicated. Wakaki et al. [6] found an IgG antibody directed against renal tubular cells in one patient that disappeared with improvement in kidney disease suggesting that there might be a common antigen in the eye and kidney resulting in the typical disease presentation. Like other autoimmune diseases, it is more common in females. Although more common in adolescence, it can present in older ages as well. Autoantibodies (ANA, ds‐DNA, ANCA and rheumatoid factor) may rarely be positive and there might be hypergammaglobulinemia suggesting activation of the immune system. TINU could be triggered by drugs and infections, but no triggering factor is identified in most of the cases. Among drugs, NSAIDs, antibiotics and the Chinese herb Goreisan [7] have been implicated. Various infections that have been linked with TINU include Epstein–Barr virus [8], reactivation of herpes zoster [9] and chlamydia [10]. There is some evidence of genetic predisposition as supported by case reports of TINU in siblings [11, 12], monozygotic twins (both had interstitial nephritis but only one developed uveitis) and a mother and son [13, 14] Some human leukocyte antigen (HLA) phenotypes have also been associated with TINU. Levinson and colleagues identified that haplotype HLA‐DQA1*01/DQB1*05/DRB1*01 was more frequently observed in patients with TINU compared to the general population with strongest association seen for the HLA‐DRB1*0102 allele (72.2%) [15].

Interest has recently focused on infectious triggers, particularly SARS‐CoV‐2. Sakhinia et al. [16] described four paediatric patients with TINU and positive SARS‐CoV‐2 serology despite the absence of COVID‐19 symptoms. In these children, kidney function improved with high‐dose steroids, but relapse occurred during tapering or after cessation, necessitating MMF as a steroid‐sparing agent. While we did not identify a COVID‐19 trigger in our cohort, asymptomatic SARS‐CoV‐2 infection cannot be excluded as serological testing was not routinely performed. These observations further support the need for long‐term follow‐up and consideration of steroid‐sparing therapy in relapsing disease.

There is no diagnostic blood or urine test for the diagnosis of TINU. Urinary β2‐microglobulin, a marker of tubular dysfunction, may serve as a useful non‐invasive biomarker, particularly in patients with preserved renal function [17]. Kase et al. [18] identified KL‐6 as a potential biomarker, reporting significantly higher serum levels in patients with TINU than in patients with uveitis from other causes. KL‐6 was not measured in our cohort. However, the diagnostic utility of KL‐6 remains uncertain, as subsequent studies have demonstrated overlap with other causes of tubulointerstitial nephritis, limiting its specificity. Similarly, Tan et al. identified elevated anti‐modified C‐reactive protein (mCRP) autoantibodies in patients with TINU and proposed mCRP as a potential shared autoantigen linking renal and ocular inflammation [19]. Although promising, mCRP testing is not routinely available in clinical practice and evidence remains limited; therefore, further validation studies are required before incorporation into routine diagnostic algorithms.

Kidney biopsy should be considered in all patients with suspected diagnosis of TINU. Characteristic light microscopy findings include tubulitis and interstitial inflammation predominantly composed of lymphocytes with fewer macrophages and plasma cells. There may be prominent eosinophilic infiltrates at initial presentation. Interstitial granulomas can also occur. With disease progression, interstitial fibrosis sets in with less inflammatory infiltrate. The degree of tubular atrophy is quite marked in TINU compared to other aetiologies of interstitial nephritis. Immunofluorescence is usually negative, and electron microscopy is also non‐contributory.

A combination of renal disease and uveitis can be seen in a number of auto‐immune and infectious conditions such as Sjögren syndrome, sarcoidosis, systemic lupus erythematosus, granulomatosis with polyangiitis, Behçet disease, tuberculosis, syphilis, Epstein–Barr virus‐associated infectious mononucleosis, toxoplasmosis, brucellosis and fungal infections such as histoplasmosis. Thus, TINU is a diagnosis of exclusion; appropriate imaging and serological/immunological tests should be done to rule out other conditions.

First line treatment is systemic ± topical steroids along with cycloplegics. As it's an uncommon disease, there is no consensus/guideline regarding optimum dose and duration of steroid therapy. Prognosis is good in most cases with complete recovery of kidney function, but a fraction of patients may have relapsing disease requiring second line therapy and may progress to CKD.

## 5 Conclusion

A diagnosis of TINU should be considered in any patient presenting with acute kidney injury and ocular inflammation with negative renal immunology. Similarly, ophthalmologists should assess renal function in patients with uveitis, particularly when accompanied by constitutional symptoms. Early recognition, biopsy confirmation and prompt corticosteroid therapy can lead to favourable renal and ocular outcomes, though vigilance for relapses and CKD progression remains essential.

Our case series provides several distinctive contributions to the existing TINU literature. We report one of the largest single centre studies and longest median follow‐up durations (44 months) among biopsy‐proven cohorts, enabling evaluation of long‐term outcomes. While most published data are paediatric‐dominant, our cohort spans a broad age range (15–67 years) with an adult predominance, demonstrating that TINU is not confined to younger populations. Although the small sample size may have influenced the age distribution, delayed recognition of renal disease may also have contributed to older age at diagnosis in some patients.

Renal relapse occurred in 60% of patients, markedly higher than prior reports (< 10%) and frequently paralleled ocular disease activity, supporting close multidisciplinary follow‐up. Higher levels of proteinuria at presentation and progression to CKD in 50% of patients despite good initial response suggest proteinuria at presentation to be a potentially negative prognostic marker. MMF was effective as a steroid‐sparing agent in relapsing disease.

Finally, our study adds value as a post–COVID‐19 era cohort without SARS‐CoV‐2 association, offering contrast to recent pandemic‐related reports and reinforcing that TINU remains an independent autoimmune entity.

## 6 Key Learning Points and Associated Clinical Implications Identified in Our Cohort

**Table:** 

Learning point	Clinical implications
Proteinuria can be present in interstitial nephritis	The presence of proteinuria carries a higher likelihood of development of CKD.
Long‐term monitoring is essential	Patients with TINU may experience relapse or CKD progression despite apparent remission, supporting the need for prolonged follow‐up.
Renal relapse is under‐recognised	More than half of patients relapsed, and renal activity often mirrored ocular disease, highlighting the importance of synchronised nephrology–ophthalmology surveillance.
Risk stratification matters	Female sex and younger age were associated with increased recurrence risk, suggesting subgroups who may benefit from closer monitoring or earlier immunosuppression.
Mycophenolate is an effective steroid‐sparing option	Mycophenolate mofetil provided effective disease control in relapsing or steroid‐dependent cases, supporting its use as second‐line therapy.
TINU should be actively sought	Patients with unexplained AKI and negative immunology should be evaluated for TINU; conversely, uveitis with systemic features warrants renal assessment.

## Author Contributions

W.A. collected and interpreted the data, prepared the abstract, edited the manuscript and approved the final manuscript. N.A. collected clinical data including patient demographics and results, drafted the initial manuscript and approved the final manuscript. C.C. supervised the study, critically revised and edited the manuscript and approved the final manuscript. S.S. revised and edited the manuscript and approved the final manuscript. D.P. revised and edited the manuscript and approved the final manuscript. J.C. provided the biopsy images and approved the final manuscript.

## Ethics Statement

This study was observational in nature, and no interventions were performed as part of the research. All data were collected as part of routine clinical practice, with no direct involvement or alteration of patient management. Therefore, informed consent was not required. Patient confidentiality was maintained throughout the study, and all data were anonymised prior to analysis.

## Conflicts of Interest

The authors declare no conflicts of interest.

## Data Availability

The data that support the findings of this study are available from the corresponding author upon reasonable request.
